# The Intersection of Cardiology and Sleep Medicine: Impact of Sleep-Disordered Breathing on Heart Failure Outcomes

**DOI:** 10.3390/jcm15145451

**Published:** 2026-07-12

**Authors:** Ivana Huljev Šipoš, Mirna Vergles, Kristina Lalić, Ivan Marasović, Petra Grubić Rotkvić, Petar Gulin, Zrinka Čolak Romić, Ana Đuzel Čokljat, Goran Batinjan, Kristijan Šipoš

**Affiliations:** 1 Department of Pulmonology, University Hospital Dubrava, Avenija Gojka Šuška 6, 10000 Zagreb, Croatia; mirna_korica@yahoo.com (M.V.); kristinalalic2@gmail.com (K.L.); imarasovic94@gmail.com (I.M.); 2 Department of Cardiology, University Hospital Centre Zagreb, Kišpatićeva 12, 10000 Zagreb, Croatia; petra.grubic84@gmail.com; 3 Department of Otorhinolaryngology and Head and Neck Surgery, University Hospital Dubrava, Avenija Gojka Šuška 6, 10000 Zagreb, Croatia; gulinpetar@gmail.com; 4 Department of Neurology, University Hospital Dubrava, Avenija Gojka Šuška 6, 10000 Zagreb, Croatia; zcr0410@gmail.com; 5 Department of Internal Medicine, General Hospital Dubrovnik, Roka Misetica 2, 20000 Dubrovnik, Croatia; ana29.djuzel@gmail.com; 6 Department of Maxillofacial and Oral Surgery, University Hospital Dubrava, Avenija Gojka Šuška 6, 10000 Zagreb, Croatia; gbatinjan@sfzg.hr; 7 Department of Cardiology, Magdalena Clinic for Cardiovascular Disease, 49217 Krapinske Toplice, Croatia

**Keywords:** obstructive sleep apnea, central sleep apnea, heart failure, hipoxic burden

## Abstract

Sleep-disordered breathing (SDB)—including obstructive sleep apnea (OSA) and central sleep apnea (CSA)—is highly prevalent among individuals with heart failure (HF), yet frequently underdiagnosed. Increasing evidence shows that SDB and HF interact in a vicious cycle: recurrent hypoxic episodes, fluctuations in intrathoracic pressure, and autonomic dysregulation further strain cardiac function, while HF itself predisposes to the development of apnea. This interplay worsens arrhythmia burden, symptom severity, and long-term prognosis. This review will explore how OSA and CSA influence HF progression and outcomes, elucidate the underlying mechanisms linking these conditions, and discuss diagnostic challenges—from simple screening questionnaires to advanced telemonitoring approaches. We will critically evaluate current therapeutic options, including continuous positive airway pressure (CPAP), adaptive servo-ventilation (ASV), and emerging technologies, in light of major clinical trials and guideline recommendations. Finally, we will highlight the importance of a multidisciplinary, patient-centered approach to improve outcomes in this complex patient population.

## 1. Introduction

Heart failure (HF) remains one of the leading causes of cardiovascular morbidity, mortality, and recurrent hospitalization worldwide despite major advances in pharmacological and device-based therapies [1]. Over the past decade, growing attention focused on comorbid conditions that may substantially influence disease progression and long-term outcomes in patients with HF, among which sleep-disordered breathing (SDB) has emerged as one of the most clinically relevant yet persistently underdiagnosed entities [2].

Sleep-disordered breathing encompasses a spectrum of respiratory abnormalities during sleep, primarily obstructive sleep apnea (OSA) and central sleep apnea (CSA). Current evidence suggests that some form of SDB may be present in approximately 50–70% of patients with HF, although reported prevalence varies according to the population studied and diagnostic methodology [2,3]. Despite this remarkably high prevalence, SDB frequently remains unrecognized in routine cardiology practice because its symptoms often overlap with manifestations of HF itself, while access to comprehensive sleep diagnostics remains limited in many healthcare systems [4].

The interaction between HF and SDB is highly complex and bidirectional. Recurrent apneic episodes induce intermittent hypoxia, sympathetic activation, oxidative stress, systemic inflammation, and endothelial dysfunction, thereby contributing to adverse myocardial remodeling and progression of cardiovascular disease [5]. Simultaneously, HF itself may predispose patients to the development of sleep apnea, particularly CSA and Cheyne–Stokes respiration, through mechanisms involving pulmonary congestion, fluid redistribution, prolonged circulation time, and impaired ventilatory control stability [5,6].

Clinically, the coexistence of HF and SDB has been associated with increased arrhythmia burden, impaired functional capacity, reduced quality of life, more frequent hospitalizations, and higher mortality [7]. Nevertheless, SDB remains substantially underdiagnosed and undertreated in HF populations, partly because traditional screening strategies often perform poorly in these patients and symptom presentation is frequently atypical.

Given the growing recognition of the close interaction between HF and SDB, this review aims to summarize current evidence regarding epidemiology, pathophysiological mechanisms, diagnostic challenges, prognostic implications, and therapeutic approaches in patients with coexisting HF and sleep-disordered breathing. Particular emphasis is placed on individualized patient phenotyping, critical interpretation of recent landmark clinical trials, and the need for multidisciplinary collaboration between cardiology and sleep medicine.

### Literature Search

This narrative review was based on a comprehensive literature search conducted using PubMed/MEDLINE, Scopus, and Google Scholar. Publications from January 1990 to September 2025 were considered, with particular emphasis on recent clinical guidelines, randomized controlled trials, systematic reviews, meta-analyses, and landmark studies addressing the relationship between sleep-disordered breathing and heart failure. Search terms included combinations of heart failure, sleep-disordered breathing, obstructive sleep apnea, central sleep apnea, Cheyne–Stokes respiration, continuous positive airway pressure, adaptive servo-ventilation, hypoxic burden, telemonitoring, heart failure outcomes, and cardiac arrhythmias. Additional relevant articles were identified through manual screening of reference lists of selected publications. Articles were selected based on their relevance to the scope of this narrative review, with priority given to high-quality evidence and contemporary international guidelines.

## 2. Epidemiology and Clinical Significance of Sleep-Disordered Breathing in Patients with Heart Failure

Heart failure is commonly classified according to left ventricular ejection fraction (LVEF) into heart failure with reduced ejection fraction (HFrEF), mildly reduced ejection fraction (HFmrEF), and preserved ejection fraction (HFpEF). Although major advances in pharmacological and device-based HF therapy have substantially improved survival, particularly in patients with HFrEF through modulation of maladaptive neurohumoral pathways, therapeutic progress in HFpEF has remained considerably more modest [1]. As the burden of HF continues to increase in an aging population, sleep-disordered breathing has emerged as an increasingly important and highly prevalent comorbidity across the entire spectrum of HF phenotypes. Most studies report that between 50% and 70% of patients with HF exhibit some form of SDB, with OSA and CSA representing the dominant phenotypes [2,3,8,9]. Notably, prevalence estimates vary considerably depending on patient selection, disease severity, and diagnostic criteria.

### 2.1. Prevalence of OSA and CSA Across Different Heart Failure Phenotypes

In patients with HFrEF, CSA—most commonly manifesting as Cheyne–Stokes respiration—has traditionally been considered the predominant form of SDB [5,10]. However, more recent data suggest that OSA may actually represent the most frequent phenotype across all major HF categories, particularly in patients with obesity, diabetes, and metabolic dysfunction [11,12]. Findings from the SchlaHF registry demonstrated that OSA prevalence exceeded that of CSA in most analyzed HF cohorts, including HFrEF, HFmrEF, and HFpEF populations [11].

Among patients with HFpEF, OSA appears particularly common, likely reflecting the high prevalence of obesity, hypertension, metabolic syndrome, and diastolic dysfunction characteristic of this phenotype [11,13,14,15]. Several studies have reported OSA prevalence exceeding 60% in HFpEF populations, although CSA also remains clinically relevant because of its association with adverse cardiovascular outcomes [11,16].

Notably, mixed forms of SDB frequently occur in patients with chronic HF, with transitions between obstructive and central respiratory events observed during the same night. These dynamic changes appear closely linked to fluctuations in cardiac function, circulation time, and arterial carbon dioxide levels [17]. Progressive reductions in PaCO_2_ together with worsening circulatory delay may facilitate transition from OSA toward CSA, emphasizing the dynamic and heterogeneous nature of SDB in HF populations.

### 2.2. Risk Factors and Predisposing Mechanisms

The development of SDB in HF results from the interaction between traditional sleep apnea risk factors and HF-specific pathophysiological mechanisms. Obesity, male sex, and advanced age remain important predictors of OSA in patients with HF [18,19]. In addition, HF-related fluid retention and nocturnal rostral fluid redistribution additionally increase upper airway collapsibility and contribute to obstructive respiratory events during sleep [20,21].

In CSA, reduced cardiac output, prolonged circulation time, pulmonary congestion, and enhanced chemoreceptor sensitivity to carbon dioxide promote instability of ventilatory control [4,5,6,22,23]. Lower awake PaCO_2_ levels further increase susceptibility to central apneas by narrowing the difference between eupneic and apneic carbon dioxide thresholds [22].

A central concept underlying CSA pathophysiology is increased loop gain, reflecting instability of the ventilatory control system. Loop gain includes controller gain (chemoreceptor responsiveness), plant gain (efficiency of pulmonary gas exchange), and circulation-related delay between lungs and chemoreceptors.

Increased chemosensitivity and prolonged circulation time amplify ventilatory oscillations, predisposing patients to unstable periodic breathing and the development of Cheyne–Stokes respiration. Additional stimulation of pulmonary and respiratory muscle receptors by fluid accumulation within the lungs and interstitium further augments minute ventilation, thereby contributing to ventilatory instability [5,6,22,23]. An important characteristic of Cheyne–Stokes respiration is its prolonged cycle duration, typically ranging between 45 and 90 s, reflecting delayed circulatory feedback and prolonged circulation time in these patients [5,6,22,23].

### 2.3. Clinical Significance and Prognostic Implications

The presence of SDB in HF has consistently been associated with poorer clinical outcomes. Numerous observational studies have linked both OSA and CSA with increased mortality risk and more frequent HF-related hospitalizations [7,8]. In particular, nocturnal hypoxemia appears to represent a strong prognostic marker independent of the apnea–hypopnea index itself [8].

Sleep-disordered breathing also contributes to increased arrhythmogenic risk, including atrial fibrillation, ventricular arrhythmias, and sudden cardiac death [24,25]. Among patients with implantable cardioverter-defibrillators, coexistence of SDB has been associated with a greater number of appropriate device therapies and shocks [26].

Interestingly, data from large longitudinal cohorts suggest that CSA may carry worse prognosis than OSA, especially in patients with advanced HFrEF [27]. Despite these observations, considerable heterogeneity exists across studies, and growing evidence suggests that the cumulative burden of nocturnal hypoxemia may be more prognostically relevant than apnea subtype alone [8,28].

Collectively, these findings support the need for more precise phenotyping of patients with SDB and HF, extending beyond conventional classification based solely on respiratory event counts.

## 3. Pathophysiological Mechanisms Linking Sleep-Disordered Breathing and Heart Failure

The relationship between sleep-disordered breathing (SDB) and heart failure (HF) reflects a complex bidirectional interaction involving hemodynamic, respiratory, neurohumoral, inflammatory, and metabolic mechanisms. Although obstructive sleep apnea (OSA) and central sleep apnea (CSA) differ in their primary pathophysiological mechanisms, both disorders are characterized by recurrent episodes of oxygen desaturation and reoxygenation, sleep fragmentation, autonomic dysregulation, and oscillatory changes in intrathoracic pressure (Table 1). Together, these disturbances impose additional stress on an already compromised cardiovascular system and may contribute to progression of both structural and functional cardiac abnormalities characteristic of HF [29,30,31,32,33,34].

### 3.1. Obstructive Sleep Apnea: Mechanical and Autonomic Cardiac Stress

In obstructive sleep apnea, recurrent collapse of the upper airway occurs during sleep despite preserved respiratory effort. Consequently, episodes of complete or partial airway obstruction develop, accompanied by intermittent hypoxemia, hypercapnia, sleep fragmentation with recurrent arousals, and pronounced swings in negative intrathoracic pressure [29,31,35].

Markedly negative inspiratory pressures increase left ventricular transmural pressure, thereby elevating afterload and reducing stroke volume. At the same time, enhanced venous return to the right heart may lead to interventricular septal shift, further impairing left ventricular filling and contributing to an additional decline in cardiac output. In patients with HF, these repetitive hemodynamic disturbances may worsen both systolic and diastolic ventricular function and increase the likelihood of pulmonary congestion [5,21,22,29,33].

These hemodynamic alterations are further aggravated by recurrent intermittent hypoxemia, which increases myocardial oxygen demand, impairs coronary perfusion, and may contribute to myocardial ischemia and progressive deterioration of cardiac function [36,37,38,39,40,41,42]. Treatment with continuous positive airway pressure (CPAP) has been shown to attenuate these adverse overnight hemodynamic effects in patients with HFrEF.

Although snoring is frequently present in patients with OSA and HFrEF, excessive daytime sleepiness is often less pronounced than in the general OSA population [43]. Patients with HF may exhibit lower Epworth Sleepiness Scale scores despite prolonged sleep latency and reduced total sleep duration, possibly due to persistent sympathetic overactivation and its sustained alerting effect [44,45,46,47]. The coexistence of OSA and HFrEF is likely to further amplify sympathetic activity and cardiovascular stress.

Recurrent cycles of intermittent hypoxia and reoxygenation in OSA promote oxidative stress, endothelial dysfunction, and systemic inflammation [12]. These processes are characterized by increased production of reactive oxygen species, reduced bioavailability of nitric oxide, and activation of multiple inflammatory and vasoconstrictive pathways, all of which contribute to arterial stiffness, endothelial injury, arithmias and adverse myocardial remodeling. At the same time, intermittent hypoxia and hypercapnia stimulate both peripheral and central chemoreceptors, resulting in sustained sympathetic nervous system activation [29,31,35,41,42,48].

In addition, repetitive arousals at the termination of apneic events are accompanied by abrupt sympathetic surges and withdrawal of parasympathetic activity, leading to transient elevations in blood pressure and heart rate. Importantly, these autonomic disturbances may extend into wakefulness, thereby maintaining persistent sympathetic hyperactivity even during daytime. Over the long term, this chronic neurohumoral activation increases myocardial oxygen demand, elevates blood pressure and heart rate, and further contributes to the progression of cardiac dysfunction in patients with HF [35,36,41,42,43,46,49].

An additional mechanism of particular importance in patients with HF is nocturnal rostral fluid redistribution [50]. During recumbency, fluid shifts from the lower extremities toward the neck and upper body, increasing peripharyngeal tissue edema and upper airway collapsibility during sleep. This mechanism may substantially contribute to the development or worsening of OSA, particularly in patients with volume overload and systemic congestion, and may also explain the occurrence of OSA in patients who are not markedly obese [20,21,29].

### 3.2. Central Sleep Apnea: Instability of Ventilatory Control

In contrast to OSA, central sleep apnea (CSA) is characterized by a reduction or complete cessation of ventilation resulting from absent respiratory effort. In patients with HF, the most common form of CSA is Cheyne–Stokes respiration, which develops as a consequence of instability within the ventilatory control system. A central role is played by increased loop gain dynamics, including enhanced chemoreflex responsiveness to carbon dioxide, reduced arterial PaCO_2_ levels approaching the apneic threshold, prolonged circulation time, and increased plant gain related to pulmonary mechanics and gas exchange efficiency [6,22,30,32,34,51].

Loop gain reflects the overall stability of the ventilatory control system and is determined by the interaction between controller gain, plant gain, and feedback gain. Controller gain refers to the ventilatory response to changes in blood gases, particularly the sensitivity of peripheral and central chemoreceptors to reductions in arterial CO_2_, resulting in exaggerated increases in minute ventilation. Plant gain describes the change in blood gas levels in response to alterations in ventilation; in HF, pulmonary congestion and fluid accumulation within the alveolar interstitium stimulate pulmonary and respiratory muscle receptors, further augmenting ventilatory drive and predisposing to hyperventilation. Similar transient increases in ventilation may also occur during initiation of CPAP therapy, when acute alveolar expansion induces reflex hyperventilation and may occasionally contribute to treatment-emergent central sleep apnea. Feedback gain, also referred to as mixing gain, reflects the delay between changes in pulmonary gas exchange and their detection by central chemoreceptors and is therefore largely determined by circulation time. In HF, circulation time may increase from approximately 10 to nearly 30 s, thereby amplifying ventilatory oscillations and predisposing patients to periodic breathing and Cheyne–Stokes respiration.

In HF, pulmonary congestion stimulates vagal irritant receptors within the lungs, promoting hyperventilation and a subsequent reduction in PaCO_2_. During sleep, even minor transient arousals followed by several deep breaths may further lower PaCO_2_ below the apneic threshold, resulting in temporary suppression of respiratory drive and the development of central apnea. The subsequent accumulation of carbon dioxide together with progressive hypoxemia then strongly reactivates ventilation, generating the characteristic cyclical pattern of ventilatory oscillations. Prolonged circulation time additionally delays feedback between the lungs, peripheral and central chemoreceptors, and the respiratory centers, thereby further amplifying ventilatory instability and periodic breathing [6,30,32,34,51].

CSA has often been regarded primarily as a marker of advanced HF severity; however, growing accumulating evidence suggests that it may also contribute directly to disease progression rather than representing a mere epiphenomenon. Recurrent fluctuations in oxygen saturation, persistent sympathetic activation, and sleep-related cardiac rhythm disturbances associated with CSA may independently worsen hemodynamic status and adversely affect prognosis. Nevertheless, the relationship between CSA and HF remains complex, which may partly explain the heterogeneous findings observed in therapeutic studies targeting CSA [6,28,30,34].

### 3.3. Sympathetic Activation and Neurohumoral Dysfunction

Chronic sympathetic nervous system activation represents one of the central mechanisms through which both OSA and CSA adversely influence cardiovascular function. In patients with HF, in whom neurohumoral activation is already a hallmark feature of the disease, this additional sympathetic burden may further promote tachycardia, peripheral vasoconstriction, increased afterload, and myocardial electrical instability [35,36,41,42,49,52,53].

Beyond autonomic imbalance, SDB also contributes to dysregulation of the renin–angiotensin–aldosterone system (RAAS). Enhanced sympathetic activity together with repetitive hypoxic exposure stimulates renin release, sodium and water retention, and progressive fluid accumulation, thereby aggravating systemic and pulmonary congestion. These mechanisms establish an important link between SDB and volume overload, particularly in patients with advanced HF [29,49,53].

In addition, nocturnal atrial stretch associated with negative intrathoracic pressure swings and fluid redistribution may increase secretion of atrial natriuretic peptide (ANP), contributing to nocturnal polyuria and nocturia, symptoms frequently reported in patients with both OSA and HF [50].

Taken together, these findings support the concept that SDB should not be regarded merely as a coexisting respiratory disorder, but rather as an active contributor to the neurohumoral, hemodynamic, and structural progression of HF [29,35,49,52,53].

### 3.4. Inflammation, Oxidative Stress, and Endothelial Dysfunction

Intermittent hypoxia, particularly in OSA, generates a repetitive ischemia–reperfusion pattern that promotes oxidative stress and activation of inflammatory pathways. This process is associated with increased levels of systemic inflammatory mediators, impaired endothelium-dependent vasodilation, and enhanced prothrombotic activity. In patients with HF, these abnormalities may further compromise microvascular function, increase susceptibility to myocardial ischemia, and contribute to progressive ventricular remodeling [31,35,49,53].

More recent evidence suggests that the pathophysiological impact of SDB is not determined solely by the frequency of respiratory events reflected by the apnea–hypopnea index (AHI), but also by the depth and duration of oxygen desaturations, collectively referred to as hypoxic burden. Accordingly, hypoxic burden may more accurately reflect the overall biological stress imposed on the cardiovascular system and could represent a more clinically relevant predictor of cardiovascular risk than the number of respiratory events alone [54].

### 3.5. Arrhythmogenesis and Cardiac Remodeling

Sleep-disordered breathing contributes to both the development and persistence of cardiac arrhythmias through several interrelated mechanisms, including fluctuations in autonomic balance, intermittent hypoxia, changes in intrathoracic pressure, myocardial stretch, and progressive structural remodeling of the heart. In OSA, pronounced negative intrathoracic pressure swings increase mechanical stress on both atrial and ventricular walls, whereas in CSA, cyclical variations in ventilation and blood gas concentrations further destabilize myocardial electrophysiological properties [36,48,55].

These effects are particularly relevant in patients with HF, in whom the arrhythmogenic substrate is often already established due to myocardial fibrosis, chamber dilatation, impaired ventricular function, and chronic neurohumoral activation. Repetitive sympathetic surges and oxygen desaturations associated with SDB may therefore facilitate both atrial fibrillation and ventricular arrhythmias, potentially increasing the risk of sudden cardiac death and adverse cardiovascular outcomes [24,25,36,55,56].

Although the initiating mechanisms differ between OSA and CSA, both disorders ultimately exert detrimental effects on cardiac structure and function. Recurrent hemodynamic stress together with intermittent hypoxia may promote myocardial fibrosis, ventricular hypertrophy, impaired myocardial relaxation, and progressive deterioration of ventricular performance. Ultimately, these processes may contribute to adverse left ventricular remodeling, worsening diastolic dysfunction, elevation of pulmonary pressures, and decline in exercise tolerance and overall functional capacity [35,49,52,53,56].

## 4. Clinical Manifestations and Diagnostic Challenges

### 4.1. Clinical Presentation and Screening Limitations

The clinical presentation of sleep-disordered breathing (SDB) in patients with heart failure (HF) is often atypical and may therefore remain unrecognized in routine cardiology practice. In the general population, obstructive sleep apnea (OSA) is commonly suspected on the basis of loud snoring, obesity, and excessive daytime sleepiness. In patients with HF, however, this classical symptom pattern is often absent or less pronounced. Symptoms such as fatigue, reduced exercise tolerance, noctural awakenings, nocturia, and dyspnea are already common manifestations of HF itself, making it difficult to distinguish whether they originate from cardiac dysfunction, sleep disruption, or both [57,58,59].

In addition, patients with HF and concomitant OSA frequently report less subjective daytime sleepiness than expected given the severity of their sleep apnea, and the absence of excessive sleepiness should therefore not be interpreted as evidence against clinically relevant SDB in this population [45,46,57,60]. In CSA, particularly when accompanied by Cheyne–Stokes respiration, classical features such as prominent snoring may be minimal or entirely absent, thereby further reducing clinical suspicion [29,30,51,57].

Screening questionnaires may represent a useful initial step in identifying patients at increased risk of SDB; however, their diagnostic performance in HF remains limited. The Epworth Sleepiness Scale (ESS), which evaluates subjective daytime sleepiness, ranges from 0 to 24, with values above 10 generally considered indicative of excessive daytime sleepiness. Nevertheless, because hypersomnolence is frequently less pronounced in HF patients, the sensitivity of ESS for detecting clinically relevant SDB is substantially reduced [45,46,60].

Similarly, the STOP-Bang questionnaire, incorporating Snoring, Tiredness, Observed Apneas, High Blood Pressure, Body Mass Index, Age, Neck Circumference, and Gender, is widely used for OSA risk stratification. Scores range from 0 to 8, while values ≥3 generally indicate increased risk for OSA. However, its performance in HF populations is less reliable, particularly because many patients with HF—especially those with CSA—do not exhibit the typical phenotype on which the questionnaire is based [59,61].

Reuter et al. demonstrated that the ESS, STOP-Bang, and Berlin questionnaires have limited sensitivity and specificity for detecting SDB in patients with cardiovascular disease, emphasizing that negative questionnaire results cannot reliably exclude clinically relevant sleep apnea [58].

Questionnaire-based screening should be complemented by evaluation of additional clinical indicators suggestive of SDB, including nocturnal dyspnea, paroxysmal nocturnal dyspnea, resistant hypertension, atrial fibrillation, recurrent HF hospitalizations, morning headaches, witnessed apneas, fragmented sleep, and unexplained daytime fatigue or cognitive impairment. The coexistence of such findings should increase clinical suspicion and prompt further objective diagnostic evaluation using dedicated sleep studies [11,57,59,62,63].

### 4.2. Polysomnography Versus Home Sleep Apnea Testing

Overnight polysomnography (PSG) remains the gold standard for the diagnosis of sleep-disordered breathing as it provides comprehensive simultaneous assessment of respiratory events, oxygen saturation, sleep architecture, arousals, and sleep stages. In patients with HF, PSG is of particular clinical importance because it allows more accurate differentiation between obstructive and central respiratory events, an issue that is highly relevant when selecting the most appropriate therapeutic strategy [29,30,51,59].

In recent years, home sleep apnea testing (HSAT) has become increasingly used due to its greater accessibility, lower cost, and practical convenience. Nevertheless, its use in patients with HF is associated with several important limitations. Unlike full polysomnography, HSAT generally does not include electroencephalographic monitoring and therefore cannot reliably determine actual sleep duration. Consequently, the apnea–hypopnea index may be underestimated, particularly in patients with fragmented sleep or reduced sleep efficiency. Moreover, HSAT is less accurate in distinguishing obstructive from central respiratory events, which may be especially problematic in HF populations where mixed and central apnea patterns are relatively common [59,62,63].

Interpretation of nocturnal oxygen desaturation in HF patients may also be challenging because hypoxemia is not always solely attributable to apneic episodes. Pulmonary congestion, impaired gas exchange, and coexisting pulmonary disease may additionally contribute to nocturnal desaturation, thereby complicating interpretation of HSAT findings [57,62,63].

For these reasons, current recommendations generally favor polysomnography in patients with HF and other complex comorbid conditions. Although HSAT may still be a reasonable initial diagnostic approach in carefully selected patients with a high pretest probability of uncomplicated OSA, negative, inconclusive, or clinically discordant findings should prompt further evaluation with full overnight polysomnography [59,62,63].

### 4.3. The Role of Implantable Devices and Telemonitoring

Modern implantable cardiac devices, including implantable cardioverter-defibrillators (ICDs), cardiac resynchronization therapy (CRT) systems, and pacemakers, are increasingly being utilized not only for rhythm management but also for the detection of sleep-disordered breathing through algorithms based on transthoracic impedance measurements and analysis of respiratory patterns [64,65,66,67]. These technologies enable continuous long-term monitoring and may help identify patients with a high likelihood of clinically significant sleep-related breathing disturbances.

Several studies have demonstrated good agreement between device-detected respiratory events and findings obtained by standard polysomnography, particularly in patients with more severe forms of sleep apnea [64,66,67,68]. Nevertheless, despite their growing diagnostic potential, these algorithms cannot replace formal sleep studies and should be regarded primarily as adjunctive tools for screening and longitudinal monitoring rather than definitive diagnostic methods [59,64,68].

Telemonitoring has further expanded the opportunities for remote management of patients with HF by enabling earlier recognition of clinical deterioration and potentially associated respiratory abnormalities [64,69]. Continuous collection and transmission of physiological data may enable earlier therapeutic intervention and improve overall disease surveillance. However, the true clinical value of telemonitoring largely depends on its integration into a multidisciplinary care model, as well as on careful interpretation of the large amount of generated data [57,64,69].

## 5. Impact of Sleep-Disordered Breathing on Outcomes in Heart Failure

Sleep-disordered breathing (SDB) in patients with heart failure extends beyond a common comorbidity and is increasingly recognized as an important determinant of prognosis, symptom burden, quality of life, and healthcare utilization [2,27,29,33,57,70]. Both obstructive sleep apnea (OSA) and central sleep apnea (CSA) have been associated with adverse cardiovascular outcomes, although their prognostic significance appears to vary according to the underlying HF phenotype and dominant pathophysiological mechanisms [7,8,27,28,71,72,73].

In OSA, adverse cardiovascular effects are mainly related to intermittent hypoxia, negative intrathoracic pressure swings, sympathetic activation, and increased arrhythmogenic potential [12,31,35,41,48,53]. By contrast, CSA is more commonly associated with advanced HF, ventilatory instability, impaired hemodynamic status, and poorer long-term prognosis [6,10,23,28,30,32,51,72]. Accumulating evidence nevertheless suggests that prognosis cannot be explained solely by apnea subtype but also depends on cumulative nocturnal hypoxemia, sleep fragmentation, autonomic burden, and the underlying HF phenotype itself [8,11,29,32,49,54,57].

### 5.1. Mortality and Hospitalizations

Observational studies consistently suggest that untreated OSA in patients with HF is associated with increased mortality risk and more frequent HF-related hospitalizations, even after adjustment for conventional prognostic factors [7,8,27,33,70,71]. Similarly, severe nocturnal hypoxemia and a high burden of respiratory disturbances in CSA have been linked to poorer survival and recurrent HF decompensation [8,9,10,28,29,72,73]. 

Emerging evidence suggests that hypoxic burden may influence prognosis and treatment response, supporting its potential role in risk stratification beyond the apnea–hypopnea index [54,74].

Interventional trials evaluating treatment of SDB in HF have produced heterogeneous and sometimes conflicting results [28,73,74,75]. These findings are discussed in detail in Section 6.

Of particular interest is the observed relationship between hypoxic burden and therapeutic response. A secondary analysis of the ISAACC cohort demonstrated that patients with the highest hypoxic burden appeared to derive greater cardiovascular benefit from CPAP therapy, whereas no clear benefit was observed in patients with lower hypoxic burden [54,76]. These findings suggest that hypoxic burden may represent a clinically meaningful biomarker for risk stratification and individualized treatment selection; however, prospective validation is still required before it can be incorporated into routine clinical decision-making.

### 5.2. Arrhythmias (Atrial Fibrillation and Ventricular Arrhythmias)

The association between SDB and cardiac arrhythmias is particularly important in patients with HF, a population already characterized by substantial intrinsic arrhythmogenic risk. Epidemiological and mechanistic data support a strong relationship association between SDB and atrial fibrillation, ventricular ectopy, ventricular tachyarrhythmias, and sudden cardiac death [24,25,48,55,71,77,78].

Several landmark studies demonstrated a clear relationship between sleep apnea severity and nocturnal arrhythmias, while others showed that OSA is associated with increased risk of sudden cardiac death, especially in the presence of severe nocturnal hypoxemia [24,25,48]. The burden of SDB is particularly high among patients with atrial fibrillation, and untreated sleep apnea may reduce the efficacy of rhythm-control strategies while increasing recurrence rates after cardioversion or catheter ablation [48,71,77,78].

In patients with HF and implantable cardiac devices, SDB has also been associated with a higher incidence of appropriate ICD therapies and greater overall arrhythmic burden [26,55,65]. Mechanistically, OSA and CSA likely contribute to arrhythmogenesis through partially distinct pathways. In OSA, intermittent hypoxia, sympathetic surges, and repetitive intrathoracic pressure swings appear to predominate, whereas CSA is more closely linked to advanced myocardial remodeling, autonomic instability, ventilatory oscillations, and more severe underlying cardiac dysfunction [30,35,41,42,48,51,55].

Although observational evidence linking SDB with atrial and ventricular arrhythmias is highly consistent, robust data demonstrating that treatment of SDB definitively reduces major arrhythmic outcomes remain limited [48,73,77,78].

### 5.3. Functional Status and Quality of Life

Sleep-disordered breathing substantially influences symptom burden, functional capacity, and quality of life in patients with HF. Patients with coexisting SDB frequently present with worse NYHA functional class, poorer exercise tolerance, more severe nocturnal symptoms, and greater limitations in daily activities [27,33,44,45,56,57,79].

Systematic reviews consistently demonstrate that coexistence of OSA and chronic HF is associated with reduced physical performance, poorer quality of life, and less favorable cardiac functional parameters [56,71,79]. These impairments are likely to reflect the combined effects of HF severity, sleep fragmentation, intermittent hypoxemia, autonomic dysregulation, and reduced exercise capacity [31,35,49,53,56].

Interventional studies assessing treatment effects on functional outcomes have shown mixed results. In OSA, CPAP therapy may improve symptom burden, daytime alertness, and selected functional parameters [72,73,80], although improvements in overall quality of life have not been consistent across all studies. In a randomized study by Servantes et al., both exercise training and CPAP improved certain clinical measures, but improvements in quality of life were more pronounced in groups undergoing exercise-based interventions [80]. These findings highlight that outcomes in HF cannot be explained solely by normalization of respiratory indices.

In CSA, therapeutic interventions frequently improve respiratory parameters and surrogate physiological markers, but these effects have yet to consistently translate into durable improvements in quality of life or major clinical outcomes [28,72,73,75].

## 6. Therapeutic Approaches

Management of sleep-disordered breathing (SDB) in patients with heart failure remains challenging, as improvement in respiratory indices does not necessarily translate into better long-term cardiovascular outcomes. Consequently, treatment goals extend beyond simple reduction of the apnea–hypopnea index (AHI) and include improvement of symptoms, functional status, quality of life, hospitalization burden, and overall prognosis [1,2,27,28,33,57,71,72,73,75].

### 6.1. General Measures

The cornerstone of SDB management in patients with HF remains optimization of guideline-directed HF therapy together with modification of contributing risk factors [1,29,33,57,81]. Weight reduction may reduce obstructive sleep apnea severity, particularly in patients with HFpEF and obesity, although in advanced HF substantial weight loss may be difficult or even undesirable because of frailty and cardiac cachexia [18,19,33,57].

Contemporary heart failure therapies, including renin–angiotensin system inhibitors, angiotensin receptor–neprilysin inhibitors, beta-blockers, mineralocorticoid receptor antagonists, sodium–glucose cotransporter 2 inhibitors, and cardiac resynchronization therapy, may all contribute to the reduction of respiratory instability by improving cardiac function, reducing pulmonary congestion, and lowering intrathoracic pressures [1,29,33,57].

Exercise training and increased physical activity may also reduce both OSA and CSA severity, partly through attenuation of nocturnal rostral fluid redistribution from the lower extremities toward the neck and lungs during recumbency [20,21,40,49,50,80]. Several studies have shown that these benefits may occur even in the absence of significant weight reduction [40,80,81].

### 6.2. CPAP in OSA and CSA

Continuous positive airway pressure (CPAP) remains the standard first-line therapy for OSA and effectively reduces respiratory events, improves nocturnal oxygenation, and attenuates sympathetic activation [12,35,41,57,81,82]. In patients with HF, CPAP has also been associated with improvements in sleep quality, daytime symptoms, and selected measures of functional capacity and cardiac performance [9,30,44,72,80].

However, the impact of CPAP on major cardiovascular outcomes remains controversial. Randomized trials and meta-analyses in cardiovascular populations have not consistently demonstrated reductions in mortality or major adverse cardiovascular events, particularly among patients without marked daytime sleepiness [81,82]. In HF populations, available evidence suggests that therapeutic benefit may depend substantially on patient selection, adherence, and SDB phenotype [72,73,81,82].

Emerging evidence suggests that hypoxic burden may help identify patients who are more likely to benefit from CPAP therapy. A secondary analysis of the ISAACC cohort demonstrated that patients with the highest hypoxic burden appeared to derive greater cardiovascular benefit from CPAP therapy, whereas patients with lower hypoxic burden showed no clear cardiovascular benefit, with some analyses suggesting a possible increase in recurrent cardiovascular events [54,76]. 

Adherence remains a major determinant of treatment efficacy, with greater nightly CPAP use consistently associated with superior symptomatic and potentially cardiovascular benefit [72,81,82,83,84].

In patients with CSA, CPAP can reduce respiratory instability and improve oxygenation, particularly when combined with supplemental oxygen therapy [9,28,30,72]. Reduction of nocturnal hypoxemia may still be clinically meaningful even though survival benefit has not been consistently demonstrated [28,72,73].

In clinical practice, management of CSA in patients with cardiovascular disease often differs considerably from standard OSA treatment strategies. Fixed-pressure CPAP is frequently preferred over automatic modalities because excessive pressure variability may destabilize respiratory control and aggravate central respiratory events [23,51,85,86]. Excessively high pressures may also impair comfort, adherence, and ventilatory stability and can contribute to treatment-emergent central sleep apnea [23,51,85,86,87]. Although treatment-emergent central apneas often diminish spontaneously over time, pressure reduction is occasionally necessary in order to improve tolerance and reduce ventilatory instability [87].

Accordingly, contemporary management increasingly favors individualized titration strategies aimed at balancing adequate control of obstructive events with avoidance of unnecessarily high pressures. Combination therapy using mandibular advancement devices (MAD) together with CPAP is increasingly being used in patients who poorly tolerate CPAP alone or require high therapeutic pressures. By mechanically enlarging the upper airway, MAD therapy may allow the use of lower CPAP and thereby improve comfort and adherence [35,41,57,87,88,89].

Nevertheless, these observations are derived from secondary analyses and should therefore be interpreted cautiously until confirmed in prospective randomized studies.

### 6.3. Adaptive Servo-Ventilation in Central Sleep Apnea

Adaptive servo-ventilation (ASV) was originally developed as an advanced ventilatory support modality designed to stabilize periodic breathing and treat CSA, particularly in patients with HF [29,30,32,34,51,86,90,91,92]. By continuously adjusting inspiratory pressure support according to the patient’s ventilatory pattern, ASV effectively reduces central respiratory events and improves nocturnal breathing stability [28,34,51,73,86,92].

The SERVE-HF trial fundamentally altered the perception of ASV therapy in HF [28,86]. In patients with HFrEF (LVEF ≤ 45%) and predominant CSA, ASV failed to improve the primary composite cardiovascular outcome and was unexpectedly associated with increased all-cause and cardiovascular mortality [28]. However, it has been suggested that the excess mortality signal observed in SERVE-HF may have been related, at least in part, to a specific ASV device generation and algorithm no longer used in current clinical practice. Contemporary ASV systems differ substantially from earlier devices and employ more advanced ventilatory algorithms and adaptation strategies [75,86,92].

The mechanisms underlying the adverse findings observed in SERVE-HF are not yet fully understood, but proposed explanations include unfavorable hemodynamic effects of positive intrathoracic pressure and interactions between ventilatory support and unstable respiratory control mechanisms in advanced HFrEF [23,30,51,84,86].

Subsequent studies, including the ADVENT-HF trial, did not confirm the increased mortality signal associated with ASV therapy [75]. However, ADVENT-HF also failed to demonstrate reductions in mortality, cardiovascular events, or HF-related hospitalizations [75]. Nevertheless, ASV therapy was associated with improvements in sleep quality, daytime sleepiness, and several HF-related symptoms, suggesting that symptomatic and functional benefits may still be achievable despite the absence of proven prognostic improvement [73,75,79].

Consequently, the role of ASV has become increasingly personalized. More recent recommendations suggest that ASV may still be considered in selected patients with HFrEF and persistent symptomatic CSA after failure of optimized CPAP therapy [1,29,32,33,34,57,86,91,92]. In this context, CPAP failure generally refers to persistent residual central respiratory events with an AHI remaining >15 events/hour despite adequate adherence and optimized CPAP treatment for more than three months. Current recommendations therefore emphasize careful patient selection, optimization of HF therapy, and individualized assessment of potential risks and symptomatic benefits rather than universal avoidance of ASV in all patients with reduced ejection fraction [1,33,34,57,73,86,91,92].

Taken together, current evidence indicates that the role of ASV remains incompletely defined, and treatment decisions should therefore be individualized according to HF phenotype, left ventricular ejection fraction, and the predominant type of sleep-disordered breathing.

### 6.4. Other Therapeutic Approaches

#### 6.4.1. Phrenic Nerve Stimulation

Phrenic nerve stimulation has become a novel therapeutic option for patients with CSA, particularly when positive airway pressure therapies are poorly tolerated or ineffective [32,34,91,92,93,94,95]. This approach uses transvenous electrical stimulation of the phrenic nerve to induce diaphragmatic contraction during sleep and restore a more physiologic respiratory pattern.

Unlike positive airway pressure therapies, phrenic nerve stimulation directly targets the absent respiratory drive characteristic of CSA [93,95]. Clinical studies have demonstrated significant reductions in central apnea burden together with improvements in sleep quality and patient-reported quality of life [93,94,95]. Long-term follow-up data suggest relatively stable therapeutic effects over time [94]. However, despite encouraging physiological and symptomatic improvements, robust evidence demonstrating reductions in mortality or HF hospitalizations remains lacking [73,91,92,94].

#### 6.4.2. Supplemental Oxygen Therapy

Supplemental nocturnal oxygen therapy represents another frequently used strategy in patients with CSA and HF, particularly when significant nocturnal hypoxemia is present [6,30,34,51,96]. Oxygen appears to stabilize breathing by improving ventilatory stability, attenuating fluctuations in oxygen tension, and reducing hypoxia-driven ventilatory overshoot, thereby decreasing ventilatory instability and central apnea frequency [6,22,30,90,96].

By reducing intermittent hypoxemia and repetitive desaturation–reoxygenation cycles, oxygen therapy may additionally attenuate sympathetic activation and some components of cardiovascular stress associated with unstable breathing during sleep [35,41,48,54,96].

In clinical practice, supplemental oxygen is particularly relevant in patients with persistent nocturnal hypoxemia due to coexisting pulmonary disease or advanced heart failure with impaired gas exchange. In such patients, oxygen therapy may complement positive airway pressure treatment by correcting residual hypoxemia despite adequate control of respiratory events [29,33,34,56,57,91,96].

Despite favorable effects on oxygenation and respiratory stability, evidence regarding long-term cardiovascular benefit remains limited. Most studies have demonstrated improvements in nocturnal oxygenation and respiratory parameters, whereas reductions in mortality or HF-related hospitalizations have not been conclusively established [72,73,91,95].

Future randomized studies are needed to determine whether correction of nocturnal hypoxemia itself translates into meaningful improvements in long-term cardiovascular outcomes.

#### 6.4.3. Emerging Technologies and Future Directions

Several emerging therapeutic strategies are currently being explored for management of SDB in HF [32,34,57,91,92,97]. Growing attention is focused on personalized ventilatory approaches adapted to individual respiratory patterns, hemodynamic characteristics, and HF phenotype. Advances in ventilatory algorithms and adaptive pressure modulation may allow more individualized treatment strategies in the future [75,91,92,98].

Optimization of cardiac resynchronization therapy may additionally contribute to improvement of CSA by improving cardiac output and reducing ventilatory instability [2,29,33,51,56]. Digital medicine and telemonitoring technologies are also playing an increasingly important role, allowing continuous monitoring of respiratory patterns, oxygen saturation, treatment adherence, and device-derived physiological signals [64,67,69,98].

A major focus of current research is development of more precise phenotyping strategies aimed at identifying patients most likely to benefit from specific therapeutic interventions. Emerging evidence suggests that variables such as hypoxic burden, arousal burden, chemoreflex sensitivity, ventilatory instability, HF phenotype, and comorbidity profile may be more informative than apnea frequency alone when selecting optimal therapy [32,54,74,76,90,91,99].

## 7. Future Perspectives and Conclusions

The association between sleep-disordered breathing (SDB) and heart failure (HF) is now widely recognized; however, the clinical implications of this interaction remain highly complex and only partially understood [1,2,29,33,57]. Although extensive pathophysiological and epidemiological evidence strongly suggests that SDB contributes to worsening HF progression, interventional studies have not consistently demonstrated improvements in major clinical outcomes such as mortality or HF-related hospitalizations [28,72,75,82,85]. This discrepancy between strong biological plausibility and relatively modest therapeutic outcome data remains one of the central unresolved challenges in contemporary cardiopulmonary medicine. This apparent discrepancy suggests that current therapeutic strategies may not fully address the biological heterogeneity of sleep-disordered breathing in heart failure, highlighting the need for improved phenotyping and more personalized treatment approaches.

One possible explanation may lie in the marked heterogeneity of SDB itself. Obstructive and central sleep apnea likely represent distinct pathophysiological phenotypes with different prognostic significance and therapeutic implications rather than merely different respiratory event patterns [23,29,30,32,51]. In addition, patients classified as having similar disease severity according to the apnea–hypopnea index (AHI) may actually experience profoundly different physiological stress and cardiovascular risk profiles. For example, prolonged and profound desaturation events are unlikely to have the same biological consequences as short and shallow oxygen desaturations, despite comparable AHI values. These observations highlight the limitations of relying exclusively on respiratory event frequency as the dominant marker of disease severity.

The concept of hypoxic burden represents an important step toward more physiologically meaningful characterization of SDB [54,74,76]. Nevertheless, currently available metrics still fail to fully capture the complexity of cardiovascular and autonomic injury associated with sleep apnea. Future research will likely require more refined phenotyping strategies that integrate not only cumulative nocturnal hypoxemia, but also temporal and morphological characteristics of respiratory events themselves. Future research should also explore whether different hypoxemia phenotypes carry distinct cardiovascular and neurocognitive consequences.

At the same time, increasing attention is being directed toward development of multidimensional physiological indices extending beyond conventional respiratory event counting. Integration of parameters such as hypoxic burden, arousal burden, heart rate response metrics, autonomic variability markers, and other physiological signals may enable more accurate characterization of disease severity and identification of patient subgroups most likely to benefit from targeted therapeutic interventions [54,74,99,100]. However, methodological limitations and lack of standardization currently limit their implementation in routine clinical practice.

However, methodological limitations and lack of standardization currently limit their implementation in routine clinical practice.

Consequently, a considerable proportion of patients remain either undiagnosed or incompletely characterized, further complicating interpretation of clinical studies and implementation of evidence-based management strategies. Future efforts should therefore also focus on developing more accessible and clinically applicable approaches for earlier identification of SDB in patients with cardiovascular disease.

In this context, digital medicine, telemonitoring, wearable technologies, and implantable device-derived respiratory monitoring are becoming increasingly important [64,65,66,67,68,69,100]. Continuous physiological monitoring may facilitate earlier recognition of respiratory instability, improve longitudinal phenotyping, and potentially support more individualized therapeutic strategies. However, the precise role of these technologies in improving long-term cardiovascular outcomes remains to be established and requires further prospective investigation.

An additional emerging concept is the potential importance of early recognition and treatment of SDB following major cardiovascular and cerebrovascular events. It has been hypothesized that timely intervention after acute myocardial infarction, acute HF decompensation, or cerebrovascular insult could potentially attenuate ongoing sympathetic activation, reduce recurrent hypoxemic stress, and improve tissue recovery. Several prospective randomized studies investigating such early intervention strategies are currently underway and may ultimately influence future therapeutic paradigms.

Finally, management of SDB in patients with HF must remain fundamentally multidisciplinary. Close collaboration between cardiologists, pulmonologists, neurologists, and sleep medicine specialists is essential for accurate diagnosis, individualized therapeutic selection, optimization of adherence, and long-term follow-up [1,29,33,57,91,92]. Future models of care are likely to require integrated cardiopulmonary and sleep medicine pathways, with greater emphasis on early recognition, individualized phenotyping, and time-sensitive initiation of therapy in selected high-risk patient populations.

## Figures and Tables

**Table 1:** Comparative overview of the pathophysiological mechanisms, clinical characteristics, and therapeutic implications of obstructive and central sleep apnea in patients with heart failure.

Characteristic	Obstructive Sleep Apnea (OSA)	Central Sleep Apnea (CSA)
Primary mechanism	Recurrent upper airway collapse despite preserved respiratory effort	Instability of ventilatory control with absent or reduced respiratory drive
Typical breathing pattern	Obstructive apneas/hypopneas with thoracoabdominal effort	Periodic breathing with crescendo–decrescendo ventilation (Cheyne–Stokes respiration)
Predominant HF phenotype	More common in HFpEF and obesity-related HF phenotypes	More prevalent in advanced HFrEF
Main pathophysiological drivers	Upper airway collapsibility, obesity, rostral fluid shift, negative intrathoracic pressure swings	Increased loop gain, enhanced chemosensitivity, prolonged circulation time, pulmonary congestion
Intrathoracic pressure changes	Marked negative intratoracic pressure swings	Less pronounced intratoracal pressure changes
Blood gas abnormalities	Intermittent hypoxemia and hypercapnia	Oscillatory hypocapnia around the apneic threshold
Sympathetic activation	Triggered by intermittent hypoxia and recurrent arousals	Triggered by ventilatory instability and oscillatory changes in blood gases
Hemodynamic consequences	Increased LV afterload, impaired LV filling, increased myocardial oxygen demand	Ventilatory instability associated with advanced hemodynamic impairment
Typical associated comorbidities	Obesity, hypertension, diabetes, metabolic syndrome, atrial fibrillation	Advanced systolic dysfunction, prolonged circulation time, severe neurohumoral activation
Prognostic significance	Associated with increased cardiovascular morbidity and mortality	Often reflects more advanced HF and worse prognosis
Daytime sleepiness	Often less pronounced in HF than in the general OSA population	Frequently absent or minimal
Response to CPAP	Generally improves respiratory events, symptoms, sleep quality and blood pressure control	Variable; may improve selected patients but not consistently long-term outcomes
Role of ASV	No established role	Considered only in carefully selected patients with persistent CSA despite optimal therapy
Relationship to HF progression	Contributes to adverse cardiovascular remodeling and HF progression	May represent both a marker and contributor to HF progression
Arrhythmogenic potential	Associated with atrial and ventricular arrhythmias through intermittent hypoxia, sympathetic activation, and negative intrathoracic pressure swings	Associated with electrical instability and autonomic oscillations in advanced HF

## Data Availability

No new data were created or analyzed in this study. Data sharing is not applicable to this article.
